# Supplementary material for: Enhancement of Water Uptake in Composite Superabsorbents Based on Carboxymethyl Cellulose Through Porogen Incorporation and Lyophilization
Source: Gels. 2024 Dec 5;10(12):797. doi: 10.3390/gels10120797 (PMC11675662; doi:10.3390/gels10120797)
Supplement: Supplementary file 1 [file gels-10-00797-s001.zip › gels-3310014-supplementary.pdf]

# Enhancement of Water Uptake in Composite Superabsorbents Based on Carboxymethyl Cellulose through Porogen Incorporation and Lyophilization

**Maria S. Lavlinskaya and Andrey V. Sorokin**

**Supplementary materials**

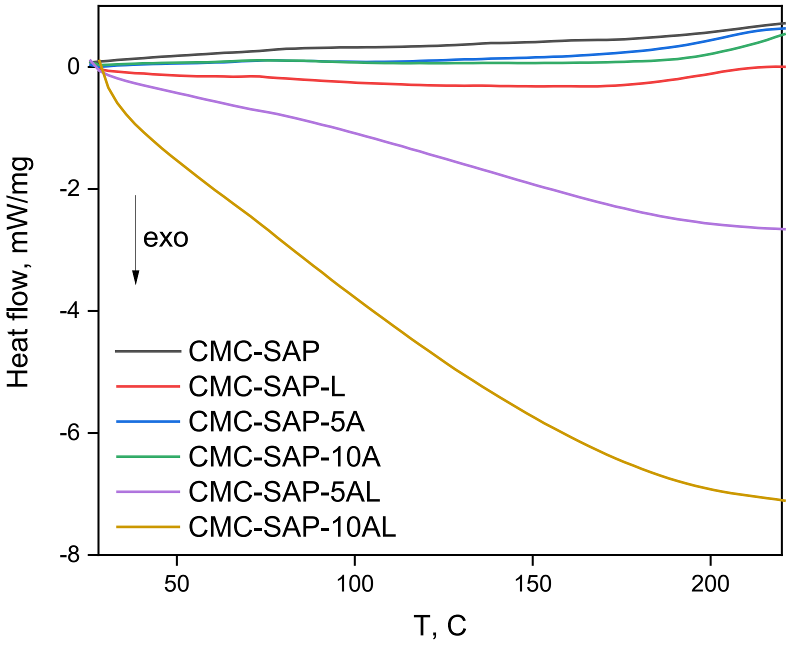


**Figure S1.** DSC profiles of the CMC-SAPs

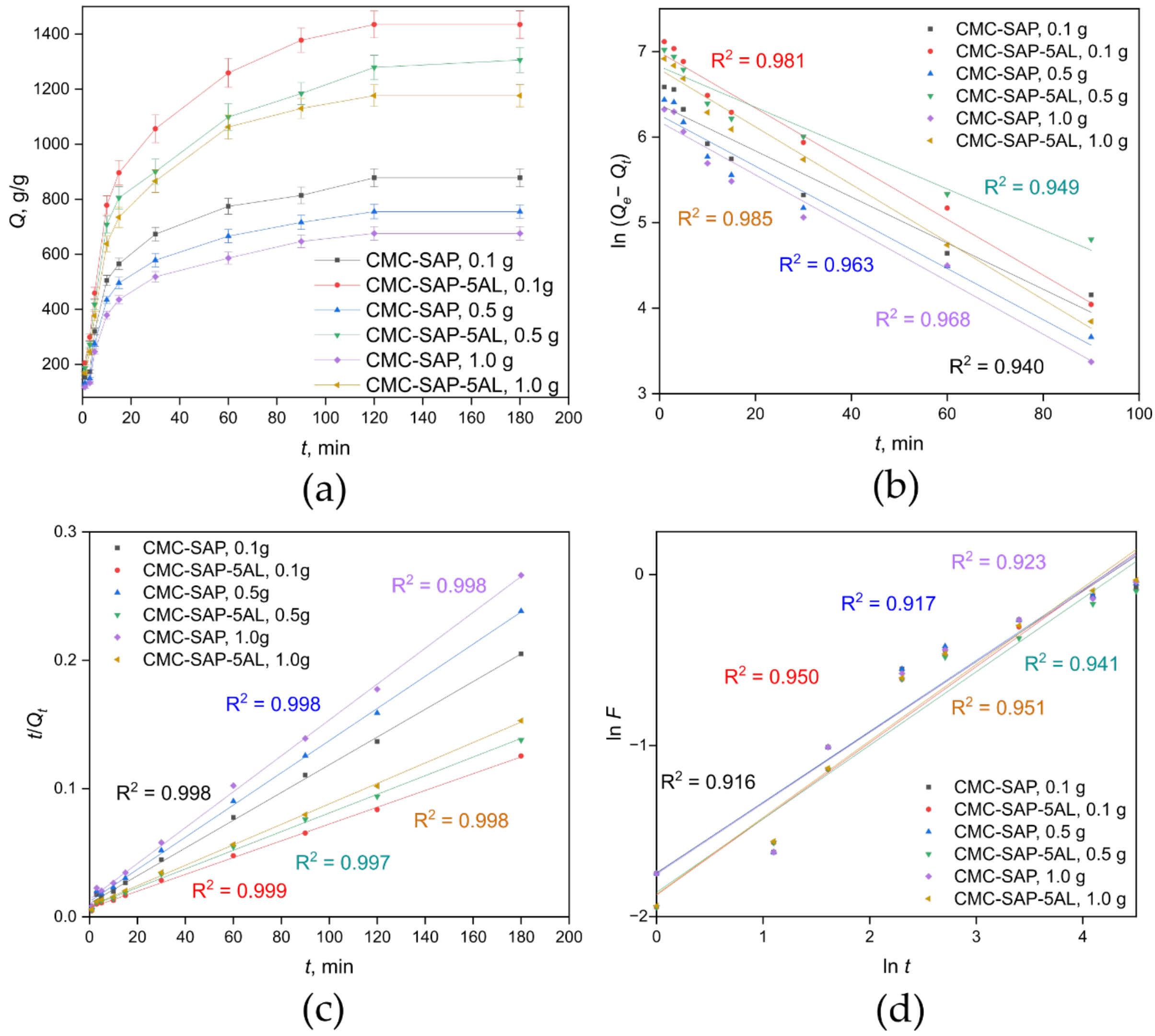


**Figure S2.** Concertation dependency of the swelling kinetics of CMC-SAPs: **(a)** swelling kinetic profiles; **(b)** swelling kinetic data processed by the pseudo-first-order model; **(c)** swelling kinetic data processed by the pseudo-second-order model; **(d)** swelling kinetic data processed by the Ritger-Peppas model.
